# Statistics on the bioactive anthocyanin/proanthocyanin products in China online sales

**DOI:** 10.1002/fsn3.2500

**Published:** 2021-07-29

**Authors:** PeiAo Zhang, Yi Li, Tianyi Wang, Zixuan Cai, Haiyan Cao, Huiying Zhang, Yubin Cao, Bo Chen, Dong Yang

**Affiliations:** ^1^ Beijing Key Laboratory of Functional Food from Plant Resources College of Food Science & Nutritional Engineering China Agricultural University Beijing China; ^2^ Xinghua Industrial Research Centre for Food Science and Human Health China Agricultural University Xinghua China; ^3^ Wenir Nutrition High‐Tech Co., Ltd Yongfeng China; ^4^ Jiangsu QingGu Foods Co., Ltd Xingdong Economic Development Zone Xinghua China

**Keywords:** Anthocyanins, food supplements, health claims, proanthocyanins

## Abstract

Due to their potential beneficial effects, anthocyanins and proanthocyanins have attracted great concern worldwide. Recently, anthocyanin/proanthocyanin‐related health products have occupied a certain proportion of the market. However, there has not been a systematical assessment on collecting and analyzing the relevant information. In this study, information of anthocyanin/proanthocyanin‐related health products on sale on the four major online shopping platforms in China has been collected from November 2020 to February 2021. A total of 144 valid samples from 91 brands were collected, among which blueberries and grape seeds are the main sources of anthocyanins and proanthocyanins, respectively. Besides, the average anthocyanins/proanthocyanins content in these products is 22.71%. Improving eyesight, anti‐asthenopia and anti‐oxidation are widely mentioned among the anthocyanin‐related products, while more proanthocyanin‐related products declare for anti‐oxidation, whitening & spot lighting, and delay of skin aging & repairing skin damage effects. Among the products, 77.78% are capsules and tablets, and the average unit price of anthocyanins/proanthocyanins is $ 5.26/g. Data analysis shows that searching for high‐quality raw materials, researching on the varieties and content of anthocyanins/proanthocyanins, focusing on the intake of specific population, and exploring better storage forms of anthocyanins/proanthocyanins may be important field in the future to promote the development of the anthocyanin/proanthocyanin‐related health products.

## Graphical abstract

We collected the information of anthocyanin/proanthocyanin‐related health products on sale on the four major online shopping platforms in China. A total of 144 valid samples from 91 brands were collected and analyzed including their sources of anthocyanin/proanthocyanin, the declared health effects, their pharmaceutical form, unit price, and custom reviews.

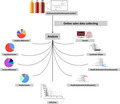

## 1 INTRODUCTION

Anthocyanins are colored, water‐soluble chemicals responsible for the bright attractive colors ranging from red‐orange to blue‐violet in plant organs such as fruits, flowers, and leaves (Liang et al., 2021). Berries, currants, grapes, and some tropical fruits are rich sources of anthocyanins (Khoo et al., 2017). Anthocyanins present red color in acidic conditions, but turn blue as the pH increases (Khoo et al., 2017). The stability of anthocyanin depends on the environmental pH, light, temperature, and structure (Laleh et al., 2005). Belonging to the flavonoids, anthocyanins are formed by modification of anthocyanidins, with two benzene rings linked by a linear three carbon chain (Liang et al., 2021; Wilska, 2007). Most identified anthocyanins are based on the six predominant anthocyanidins, which are cyanidin, delphinidin, pelargonidin, peonidin, petunidin, and malvidin (He & Giusti, 2010; Khoo et al., 2017).

Scientific studies show that anthocyanins possess antioxidative, anticancer, anti‐obesity, antidiabetic and antimicrobial effects, as well as improvement of visual and neurological health (Cisowska et al., 2011; Henriques et al., 2020; Huang et al., 2018; Lee et al., 2017; Lin et al., 2017; Reis et al., 2016; Turrini et al., 2017). Besides, there is no reported toxicity of anthocyanins in current human intervention studies (Wallace & Giusti, 2015). Numerous extraction methods have been used to extract anthocyanins. The classic method is known as solid–liquid extraction, which is based on the use of organic solvents. In addition, some modern technologies have been applied to extract anthocyanins, including supercritical fluid extraction, ultrasound‐assisted extraction, pressurized liquid extraction, microwave‐assisted extraction and ohmic heating‐assisted extraction (Fernandes et al., 2020; Hsieh‐Lo et al., 2020; Jafari et al., 2019; Liazid et al., 2011; Lopez et al., 2018; Loypimai et al., 2015; Maran et al., 2014; Petersson et al., 2010; Seabra et al., 2010; Silva et al., 2017; Vatai et al., 2009; Vieira et al., 2013).

Proanthocyanins, also known as condensed tannins, are phytonutrients formed by the condensation of flavan‐3‐ol units including catechins, epicatechins, and their gallic acid esters (Hummer & Schreier, 2008; Nie & Sturzenbaum, 2019). Colorless in their original unoxidized form, proanthocyanins obtained their name from the characteristic oxidative depolymerization reaction in acidic medium, which produces colored anthocyanidins (Hummer & Schreier, 2008). Proanthocyanins are widely distributed in the plant kingdom, including barks, leaves, flowers, fruits, and seeds of plants, and are abundant in edible plants like vegetables, fruit, nuts, and spices (Rodriguez‐Perez et al., 2019). Proanthocyanins are sensitive to oxygen, light, acid and alkaline, and polyphenol oxidase can also rapidly reduce the content of proanthocyanins in plant tissues (Shi et al., 2005).

According to the degree of polymerization, proanthocyanins can be divided into oligomers and polymers. Oligomers are formed by 2–10 flavan‐3‐ol units, while polymers may be composed of more than 10 units (Zeng et al., 2020). In addition to the degree of polymerization, proanthocyanins can be divided into A‐type and B‐type proanthocyanins according to the connection mode between flavan‐3‐ol units (Zeng et al., 2020). In the B‐type proanthocyanins, flavan‐3‐ol units are mainly linked through C4‐C8 bonds or sometimes through C4‐C6 bonds, in which both the linkages are called B‐type linkages. When there is an extra ether bond mainly formed between C2 and C7, this linkage is called A‐type linkage, and the compounds are thus called A‐type proanthocyanins (Hummer & Schreier, 2008). The A‐type linkage is less common in proanthocyanins, while B‐type proanthocyanins are widely present in many plant foods (Feng et al., 2016; Nuto, 2007). Under certain conditions, A‐type and B‐type proanthocyanins can be transformed into each other (Zeng et al., 2020). Recently, studies on proanthocyanins have attracted lots of attention since a number of pharmacological effects of proanthocyanins have been reported, including anti‐oxidative, anti‐inflammation and antimicrobial, as well as neuroprotection and metabolism‐regulation activities (Denev et al., 2019; Ma et al., 2018; Niu et al., 2016; Sun et al., 2017; Tie et al., 2020; Wang et al., 2020; Wu et al., 2019; Zhang et al., 2019). Moreover, current toxicological research indicates that proanthocyanins exhibit no observable toxicological effects on organisms (Evans et al., 2014; Sano, 2017). The conventional approach used to extract proanthocyanins is the same to the method to extract anthocyanins, while some advanced techniques have been reported recently, including enzymatic treatment, microwave‐assisted extraction, ultrasound‐assisted extraction, and supercritical fluid extraction (Chen et al., 2020; Chu et al., 2019; Fernandez et al., 2015; Hollands et al., 2017; Ma et al., 2014; Nguyen et al., 2017). However, not all these advanced techniques are currently applicable to industrial production of proanthocyanin (Barba et al., 2015; Lucarini et al., 2018).

Due to their natural abundance and health benefits, anthocyanins and proanthocyanins are applied in food, pharmaceutical, and daily necessities industries. Besides, anthocyanins can also be applied as natural dyes owing to their attractive colors (Khoo et al., 2017). This paper conducted an assessment on the international and Chinese health products related to anthocyanins/proanthocyanins from four major online shopping platforms in China, aiming at obtaining these health products’ brands, source of anthocyanins/proanthocyanins, declared health effects, product categories, price and so on, and providing reference for such health product manufacturers to better position their current products and market, as well as to develop new products for the benefit of the society.

## 2 METHODS

All the health products related to anthocyanins or proanthocyanins, approved by their corresponding regulation agency, sold on the four major online shopping platforms Taobao (https://www.taobao.com/), Tmall (https://www.tmall.com/), JD.com (https://www.jd.com/), and Vipshop (https://www.vip.com/) in China have been collected from November 2020 to February 2021, including product brands, sources of anthocyanins/proanthocyanins, content of anthocyanins/proanthocyanins, declared health effects, categories, applicable population, price, and the total number of customer reviews. Customer reviews typcially emphasized the positive quality and customer experience. Information about the same product on different platforms has been merged. Statistics and figures were made with Prism software (version 8.0, GraphPad Software Inc, La Jolla, CA, USA).

The unit price of anthocyanins or proanthocyanins were calculated with the following method. The price ranges of each product were obtained and the average of each price range was used as the average price of the product (P in $). The content of anthocyanins or proanthocyanins (C in g) in each product was also obtained. The following formula was used to calculate the unit price of anthocyanins or proanthocyanin (Y): Y = P/C.

## 3 RESULTS

### 3.1 Statistics of Brands and Customer Reviews

**FIGURE 1:**
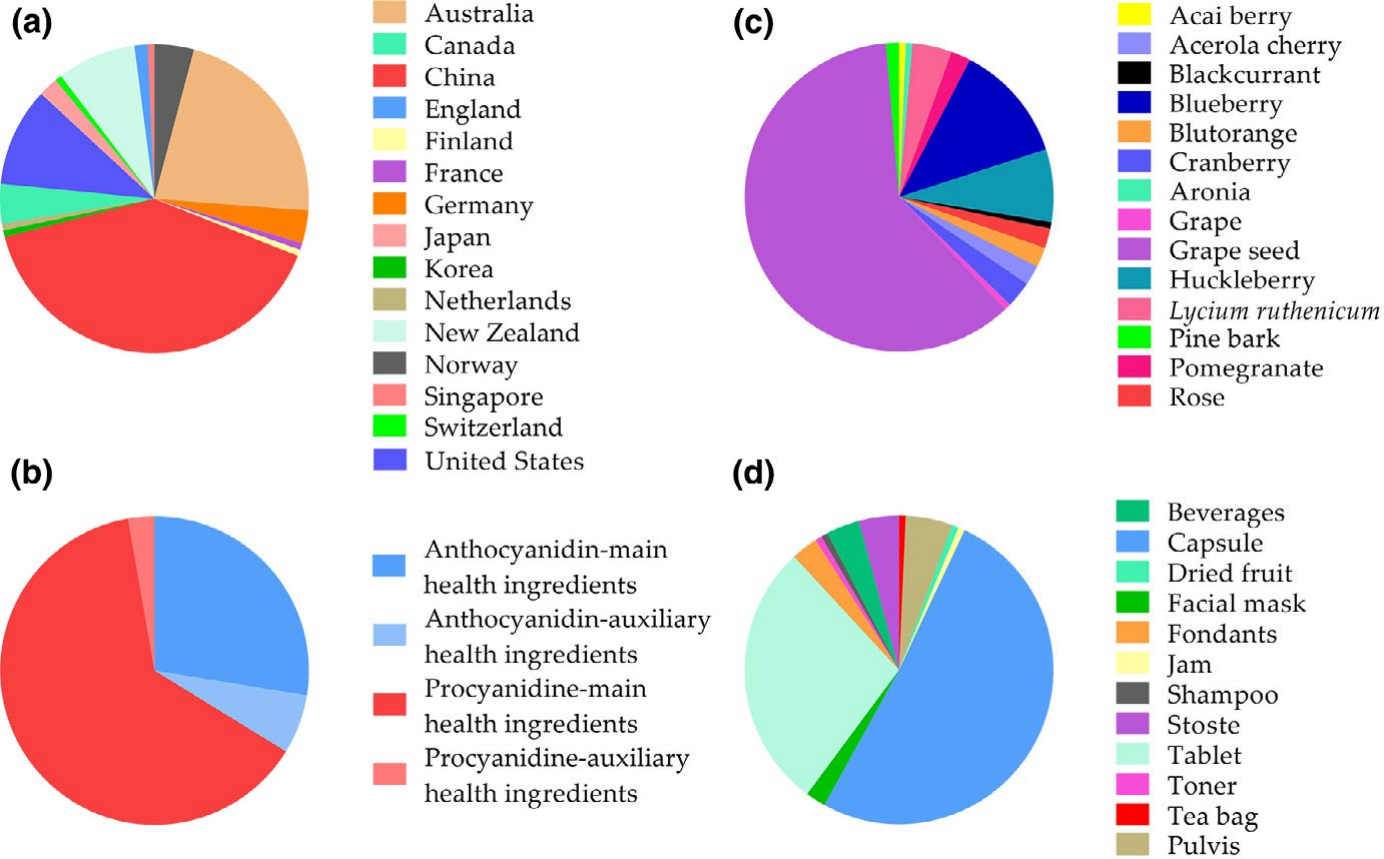
Anthocyanin/proanthocyanin product analysis. a, Pie charts of the proportion of anthocyanin/proanthocyanin products and their location. b, Pie charts of the proportion of the anthocyanin‐ or proanthocyanin‐related products. c, Pie charts of the proportion of anthocyanin/proanthocyanin‐related products with different sources. d, Pie charts of different forms of anthocyanin/proanthocyanin products

A total of 193 products related to anthocyanins and proanthocyanins were collected from these four platforms, and 144 valid samples from 91 brands were obtained after merging the same product, of which 18 brands had more than two products. These 91 brands consist of 54 international brands and 37 Chinese brands. The product location is shown in Figure 1a, in which China has the most products of 58, followed by Australia and the United States. Moreover, the total number of products from developing countries is fewer than that of developed countries.

The typical brands, corresponding product quantities and brand locations are shown in Table 1. Brands such as Swisse, HEALTHY CARE, and Tongrentang Chinese Medicine provide more products than others.

**TABLE 1:** Major anthocyanin/proanthocyanin products brands

Brand	Product quantities	Brand location
Swisse	9	Australia
HEALTHY CARE	7	Australia
Tongrentang Chinese Medicine	7	China
Xiuzheng	7	China
GO Healthy	5	New Zealand
BYHEALTH	4	China
GNC	4	America
BLACKMORES	3	Australia
Unichi	3	Australia
CONBA	3	China
Yangshengtang	3	China
Jamieson	3	Canada
Puritan's Pride	3	America
Doppelherz	2	Germany
BIOHEK	2	China
FRANIC	2	China
Esmond Natural	2	America
PipingRock	2	America
Holland&Barrett	2	England

**FIGURE 2:**
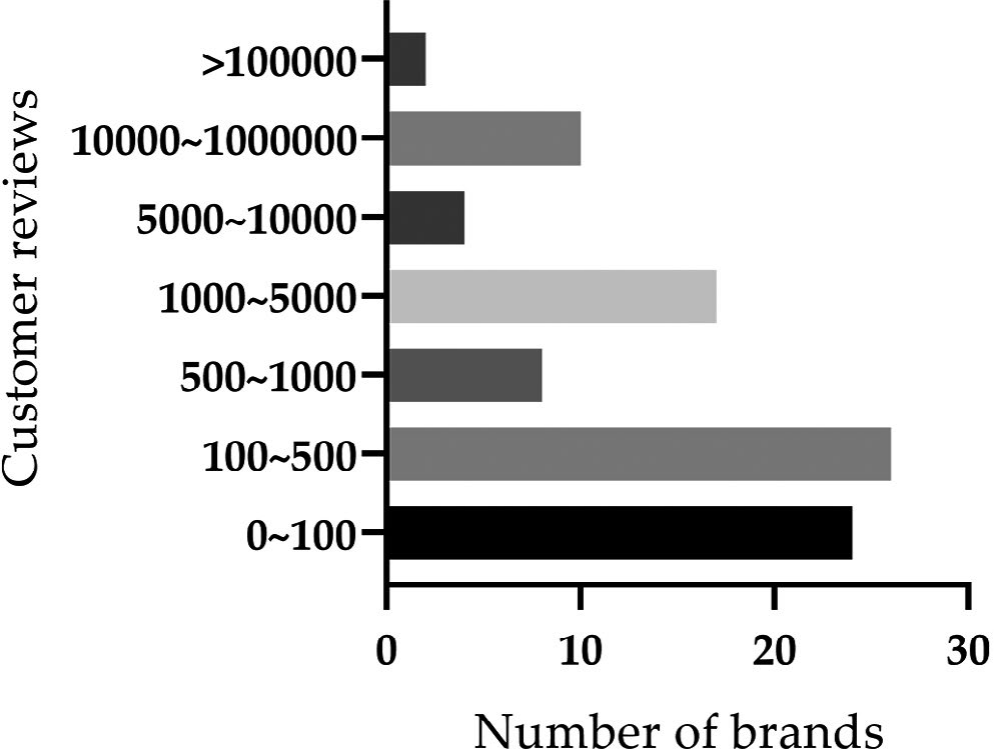
Number of brands with different customer reviews. The number of anthocyanin/proanthocyanin products bands with different number of customer reviews are listed. Customer review numbers are grouped into 0–100, 100–500, 500–1000, 1000–5000, 5000–10000, 10000–100000, more than 100000, respectively

The number of brands corresponding to different customer reviews is shown in Figure 2. There are two brands with a total of over 100,000 customer reviews, namely HEALTHY CARE and Swisse, and they are all from Australia.

### 3.2 Statistics of Products Ingredient

Among the 144 valid products, 49 are related to anthocyanins, and the other 95 are related to proanthocyanins. In addition, anthocyanins are the main health ingredients in 40 of the 49 products related to anthocyanins, while proanthocyanins are the main health ingredients in 86 of the 95 products related to proanthocyanins, as shown in Figure 1b.

The sources of anthocyanins or proanthocyanins of the 144 products were analyzed, as shown in Figure 1c. Among the 49 anthocyanin‐related products, the sources of anthocyanins are acai berry, aronia, *Lycium ruthenicum*, pomegranate, blueberry, huckleberry, blackcurrant, rose, blutorange, and acerola cherry. Among them, blueberry and huckleberry are the main sources, and the corresponding product quantities are 18 and 11, respectively. Among the 95 products related to proanthocyanins, the sources of proanthocyanins are cranberry, grape, grape seed, and pine bark. Grape seed is the main source for proanthocyanin, and there are 88 corresponding products.

Among the 144 products, 90 provided the content of anthocyanins or proanthocyanins, and 23 provided the content of extracts, that is, sources of anthocyanins or proanthocyanins. And the rest 31 did not provide either of the above two content. Besides, the content of anthocyanins or proanthocyanins in 57 of the 90 products providing the content of anthocyanins or proanthocyanins can be obtained with an average of 22.71%.

### 3.3 Declared Health Effects

**FIGURE 3:**
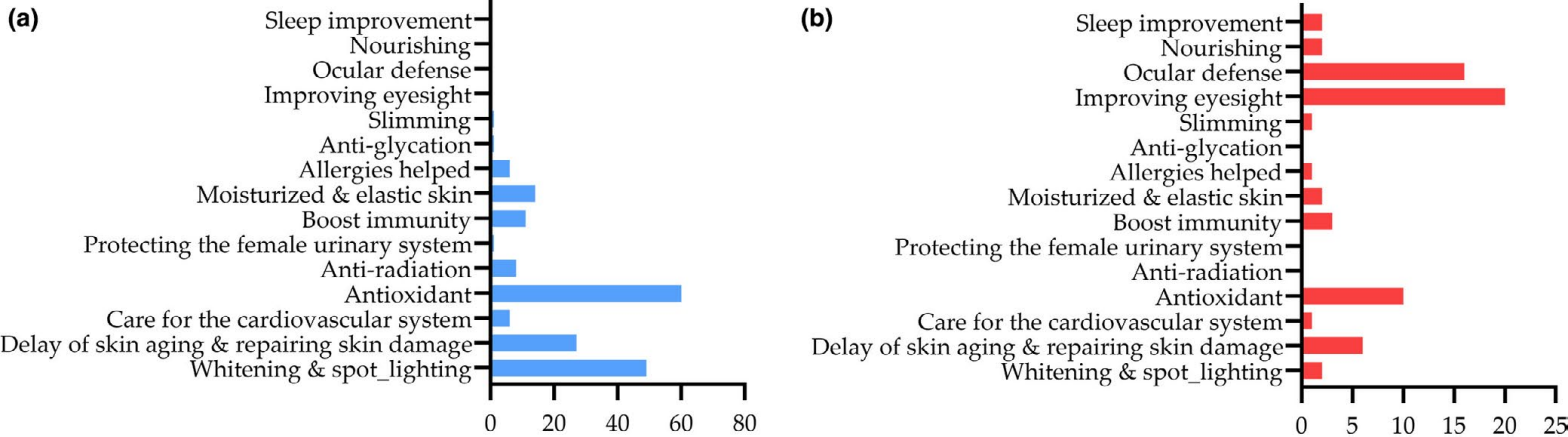
Declared health effects and the corresponding products number. a, the declared health beneficial effects of proanthocyanin. b, the declared health beneficial effects of anthocyanin

The declared health effects of all products and the corresponding number of products were analyzed, as shown in Figure 3. There are a total of 15 health effects declared, and some products declare multiple health effects. Among the anthocyanin‐related products, improving eyesight, anti‐asthenopia, and anti‐oxidation are most widely mentioned, while more proanthocyanin‐related products declare for anti‐oxidation, whitening & spot lighting and Delaying of skin aging & repairing skin damage effects. Anti‐radiation, care for the female urinary system and anti‐glycation are the unique health effects declared by proanthocyanin‐related products, while improving eyesight, ocular defense, nourishing, and improving sleep are the unique health effects declared by anthocyanin‐related products.

### 3.4 Products Forms and Applicable Population

The product forms of 144 products were analyzed, as shown in Figure 1d. The largest proportion is capsule, which accounts for 72 products. While 40 products are tablets, taking the second place as 27.78%. The applicable population of all products was also analyzed, and only two products are claimed universally applicable for the whole population. There are 130 products claimed not suitable for pregnant women, infants, and children or they must be approved by a doctor. Another eight products are dedicated for middle‐aged and elderly people, and two products are dedicated for children. The remaining two products did not mention their applicable population.

### 3.5 Unit Price

**FIGURE 4:**
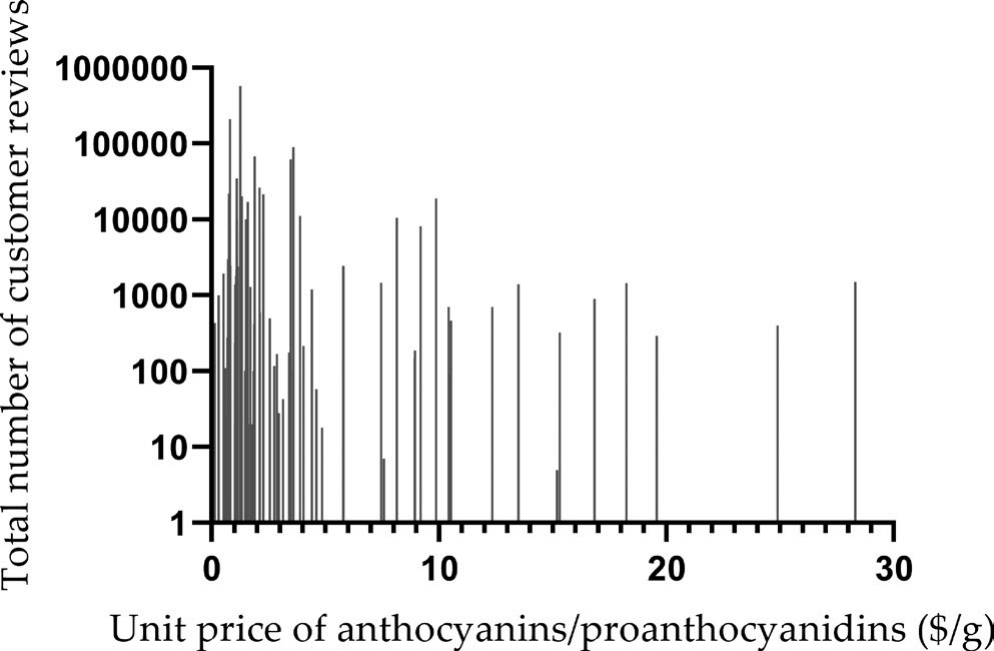
The relationship between the unit price of anthocyanins/proanthocyanins ($/g) and the total number of customer reviews

There are 84 products for which the unit price of anthocyanins or proanthocyanins (Y) can be obtained, and the average unit price is $ 5.26/g. The product with the highest unit price ($ 28.31/g) is NATURE'S CARE from Australia, while the product with the lowest unit price ($ 0.14/g) is GloryFeel from Germany. The relationship between the unit price (Y) and the total number of customer reviews is shown in Figure 4. Product prices are mostly distributed around and below the average price, and the number of products above the average price is small and scattered. Most of the total number of reviews is below 10,000 and in the range of $ 0.14–6.12. For products with more than 10,000 reviews, the price is in the range of $ 0.61–9.95. Besides, products with the unit price significantly lower or higher than the average price have less total customer reviews.

## 4 DISCUSSION

A total of 144 valid anthocyanin/proanthocyanin‐related health products were collected, brands of which are from all around the world, while mainly distributed in Australia, China, New Zealand, and the United States. There are fewer products in developing countries than in developed countries, possibly because people in developed counties focus more on healthy diet. The main sources of anthocyanins/proanthocyanins are blueberries and grape seeds, respectively, due to the high anthocyanins/proanthocyanins content of these two raw materials and the low cost of extraction. The development of high‐quality raw materials can be a research topic in the future. Most products indicated the content or source of anthocyanins/proanthocyanins in instructions, whereas did not illustrate the exact anthocyanins/proanthocyanins chemical structures, or the specific anthocyanins/proanthocyanins as the major content. Thus, more basic research should focus on studying the varieties and contents of anthocyanins/proanthocyanins in raw materials, and each of their specific health beneficial effect. Meanwhile, most products have their applicable population stated, but there are no related studies on the recommended daily intake of anthocyanins/proanthocyanins for specific population, as well as specific source of anthocyanins/proanthocyanins suitable for specific population. In terms of products forms, 77.78% of the product forms are capsules and tablets. However, it is necessary to study whether capsules and tablets are better storage forms of anthocyanins/proanthocyanins or if other formulations work better.

## 5 CONCLUSIONS

A total of 144 valid anthocyanin/proanthocyanin‐related health products have been collected, including information of product brands, source of anthocyanins/proanthocyanin, content of anthocyanins/proanthocyanin, the total number of customer reviews, the unit price, and so on. According to the data statistics, the main sources of anthocyanins/proanthocyanin are blueberries and grape seeds, respectively, and the average anthocyanin/procyanidin content obtained was 22.71%. Besides, most of the product categories are capsules and tablets, and the average unit price of anthocyanins/proanthocyanins is $ 5.26/g. Searching for high‐quality raw materials, researching on the varieties and content of anthocyanins/proanthocyanins as one aspect of quality control, optimizing on the intake of specific population (e.g. best dosage for people at different ages), and exploring better storage forms of anthocyanins/proanthocyanins would be of great value for improving health product industry related to anthocyanin/proanthocyanin.

## CONFLICT OF INTEREST

The authors declare no conflict of interest.

## AUTHOR CONTRIBUTIONS

**PeiAo Zhang:** Data curation (equal); Formal analysis (equal); Investigation (lead); Software (lead); Writing‐original draft (equal). **Yi Li:** Writing‐original draft (equal). **Tianyi Wang:** Data curation (equal); Investigation (supporting). **Zixuan Cai:** Data curation (supporting); Investigation (supporting). **Haiyan Cao:** Investigation (supporting); Resources (supporting). **Huiying Zhang:** Investigation (supporting); Resources (supporting). **Yubin Cao:** Project administration (equal); Resources (equal). **Bo Chen:** Conceptualization (supporting); Project administration (equal); Resources (equal). **Dong Yang:** Conceptualization (lead); Data curation (equal); Formal analysis (equal); Funding acquisition (lead); Methodology (lead); Project administration (equal); Resources (equal); Supervision (lead); Writing‐review & editing (lead).

## ETHICAL APPROVAL

There is no human or animal subjects involved in this study.

## Data Availability

All data generated or used in this study appear in the submitted article.
